# Sesame Lignans and Vitamin E Supplementation Improve Subjective Statuses and Anti-Oxidative Capacity in Healthy Humans With Feelings of Daily Fatigue

**DOI:** 10.5539/gjhs.v7n6p1

**Published:** 2015-03-25

**Authors:** Daisuke Takemoto, Yoko Yasutake, Namino Tomimori, Yoshiko Ono, Hiroshi Shibata, Junichi Hayashi

**Affiliations:** 1 Institute for Health Care Science, Suntory Wellness Ltd., Osaka, Japan; 2 Department of General Medicine, Kyorin University School of Medicine, Tokyo, Japan

**Keywords:** daily fatigue, oxidative stress, sesamin, vitamin E

## Abstract

Sesamin has anti-oxidative functions *in vivo*. Fatigue is caused in part by oxidative stress. We evaluated whether sesame lignans (sesamin/episesamin = 1/1, 10 mg) with vitamin E (55 mg of α-tocopherol) (SVE) could improve subjective statuses and anti-oxidative capacity in humans using questionnaires on fatigue, sleep and physical appearance, as well as low-density lipoprotein oxidation lag time. A placebo-controlled, double-blind, parallel-group study was conducted with subjects experiencing daily fatigue. After a run-in period, subjects were administered oral SVE or a placebo (P) for 8 weeks. A questionnaire regarding fatigue, sleep and physical appearance was conducted at 0, 4, and 8 weeks. Plasma low-density lipoprotein oxidation lag time was measured as an indicator of anti-oxidative capacity. The per-protocol analysis revealed significant improvements in fatigue status at 4 and 8 weeks compared to 0 weeks in both groups (p < 0.01), and sleep and physical appearance at 8 weeks compared to 0 weeks only in the SVE group (p < 0.01). There were no significant differences observed between the groups. According to the 72-subject subgroup analysis (aged 40 and over), the sleep and physical appearance significantly improved compared to the P group (p < 0.05), and fatigue status showed a tendency for improvement compared to the P group. Anti-oxidative capacity in the SVE group significantly increased compared to the P group (p < 0.01). No adverse events relating to SVE supplementation were confirmed. These results suggest SVE supplementation could safely alleviate daily fatigue and oxidative stress.

## 1. Introduction

Fatigue can be defined as the reduced ability to function and the feeling of tiredness due to physical and/or mental exhaustion. Severe cases lead to chronic fatigue syndrome (CFS), which is characterized by an abnormally prolonged period of fatigue without mechanical problems, no improvement in spite of temporary rest, and the inability to lead a healthy social life (Holmes et al., 1988; Kitani, Kuratsune, & Yamaguchi, 1992). A number of people feel occasional tiredness or continually tired and this unrecoverable, chronic feeling of fatigue has become a major social problem (Watanabe, 2008). It causes loss of vigor, motivation and youthfulness, and lowers the quality of sleep and physical appearance, resulting in reduced productivity and quality of life. Therefore, effective treatment of CFS would have a significant economic impact.

Currently, fatigue science is a popular field of research, but the molecular mechanisms underlying fatigue are not well understood due to the complicated nature of its causes. Recent studies have suggested the involvement of oxidative stress and systems such as the endocrine, metabolic, autonomic nervous system, and immune system in fatigue (Jason, Porter, Herrington, Sorenson, & Kubow, 2009; Maes & Twisk, 2010). In particular, reactive oxygen species (ROS) cause oxidative damage to proteins, lipids, and DNA, and can contribute to functional disorders in cells and tissues with reduced levels of energy (Fulle et al., 2000; Jason et al., 2009). ROS levels are related to the severity of fatigue and are very high in subjects diagnosed with CFS (Kennedy et al., 2005). These findings indicate that suppressing oxidative stress by ingesting anti-oxidative food supplements might be effective in alleviating chronic fatigue. Vitamin E (VE) is an important and powerful fat-soluble antioxidant that can inhibit lipid oxidation. Twenty-eight-week-old mice administered VE (5.0 g α-tocopherol acetate/kg diet) showed a 40% increase in median lifespan (Navarro et al., 2005). Furthermore, blood VE levels in CFS patients are low, suggesting that these patients have an increased susceptibility to oxidative stress (Miwa & Fujita, 2010).

For centuries, sesame (*Sesamum indicum* L.) has been consumed globally as a health food. Sesamin, a sesame lignan, is a major ingredient in sesame seeds and oils. It is partially isomerized to episesamin, another sesame lignan, in the refining process of sesame oil from sesame seeds. We previously reported that sesame lignans (sesamin/episesamin = 1/1) effectively inhibit vigorous exercise-induced serum lipid peroxidation in mice (Ikeda et al., 2003) and humans (Kiso, 2004), protect against alcohol-induced liver injuries (Akimoto et al., 1993), and enhance redox-regulatory (radical scavenging) activity in the livers of rats (Tada et al., 2013). However both sesamin and episesamin themselves do not have anti-oxidative properties. At the metabolic pathways, sesamin is metabolized to (1R,2S,5R,6S)-6-(3,4-dihydroxyphenyl)-2-(3,4-methylenedioxyphenyl)-3,7-dioxabicyclo[3,3,0]octane (SC-1) and (1R,2S,5R,6S)-2,6-bis(3,4-dihydroxyphenyl)-3,7-dioxabicyclo[3,3,0]octane (SC-2) through the oxidative demethylenation by metabolic enzyme, and episesamin is similarly converted to (7α,7’β,8α,8’α)-3,4-dihydroxy-3’,4’-mwthylenedioxy-7,9’:7’,9-diepoxylignane (EC-1-1), (7α,7’β,8α,8’α)-3,4- methylenedioxy-3’,4’-dihydroxy-7,9’:7’,9-diepoxylignane (EC-1-2) and (7α,7’β,8α,8’α)-3,4:3’,4’-bis(dihydroxy) -7,9’:7’,9-diepoxylignane (EC-2) as previously described (Nakai et al., 2003; Tomimori et al., 2012). These metabolites are further methylated by catechol-O-methyltransferase (COMT). The metabolites possessing the catechol group could exert potent anti-oxidative effects *in vivo* (Nakai et al., 2003), suggesting that many biological functions of sesame lignans *in vivo* could depend on the anti-oxidative activities of their metabolites.

Sesame lignans enhanced the VE activity in rats (Yamashita, Iizuka, Imai, & Namiki, 1995), and furthermore sesamin reduced the blood cholesterol level synergistically with α-tocopherol (a ratio of sesamin: α-tocopherol = 1:5 (w/w)) in rats fed a high-cholesterol diet (Rogi, Tomimori, Ono, & Kiso, 2011). Thus, the combination of sesame lignans and VE at the ratio as shown above might improve fatigue-related status and oxidative stress in people suffering from fatigue. In this study, we designed a placebo-controlled, double-blind, parallel-group study to evaluate whether the supplementation of sesame lignans with VE could improve subjective statuses corresponding to fatigue, sleep, and physical appearance, and anti-oxidative capacity in healthy humans experiencing feelings of daily fatigue.

## 2. Materials and Methods

### 2.1 Human Study Design

The study protocol conformed to the principles of the Declaration of Helsinki and was approved by the Ethics Committee of the Japanese Society of Haemorheo-Vascular Harmonization. Prior to starting the study, each prospective participant was given a full explanation of the objectives and methods of the study, and voluntary written informed consent was obtained. Subjects were selected from healthy males and females 30 to 69 years old who felt daily fatigue and were willing to participate in the study. Individuals were excluded according to the following criteria: they had a disease requiring treatment by a physician, possessed an allergy to the contents of the test foods, were pregnant or nursing, or ingested a drug or supplement daily that might impact the results of the study. Three hundred and twenty four subjects were administered placebo capsules for a 2-week run-in period. After the exclusion of 15 people based on the discretion of the study investigator, a placebo-controlled, double-blind, parallel-group study was performed with 309 subjects allocated to either the SVE group (n = 154) or placebo group (n = 155). An allocation of the subjects was performed based on the results at the start of the run-in period (–2W) so that the two groups showed no significant differences in age, sex, body mass index, severity of subjective feelings of fatigue, or such lifestyle habits as sleep time and exercise habit.

An original Physical Condition and Mood Questionnaire was prepared to assess physical and psychological fatigue and subjective mood, with reference to a previously reported questionnaire (Chalder et al., 1993; Ellis et al., 1981; Yokoyama, Araki, Kawakami, & Takeshita, 1990). We assessed 12-item questions derived from this questionnaire to evaluate the effect of SVE supplementation on subjective assessments of fatigue, sleep, and physical appearance. The questions consisted of 6 items on fatigue status (easily get tired, cannot get rid of fatigue, feeling fatigued, feeling languid, eyestrain, vigor); 3 items on sleep status (light sleeping, awaken feeling unrested, difficulty in falling asleep); and 3 items on physical appearance (skin in bad condition, poor hair luster/resilience, youthfulness). Questions were answered based on an individual’s condition during the most recent week according to a scoring system of 0 to 4: ‘never’ (0); ‘rarely’ (1); ‘sometimes’ (2); ‘very often’ (3); and ‘always’ (4). The subjects were instructed to always complete the questionnaire at home and in a physically and mentally calm state.

A physician-led interview, physiological measurements, and an evaluation of the subjective statuses using the questionnaire described above were performed at –2W, before ingesting the experimental supplements (0W), and during the 4th and 8th weeks of the experimental supplement ingestion period (4W and 8W, respectively). Blood samples were collected at the same time from the assigned subjects to assess safety and anti-oxidative capacities. The subjects were instructed to perform daily self-assessments using the questionnaire and to make no changes in their dietary habits, exercise, sleep time, or other lifestyle habits during the study.

### 2.2 Experimental Supplements

The test supplement SVE (Sesamin E Plus, Suntory Wellness Ltd.) consisted of 10 mg sesame lignans (sesamin/episesamin=1/1), VE (55 mg of α-tocopherol, 44 mg of γ-tocopherol, and 2 mg of tocotrienol), and wheat germ oil (the content of α-tocopherol is negligible) per 3 capsules. The control (placebo) supplement consisted of only wheat germ oil without active ingredients and was indistinguishable in appearance from the SVE supplement. The SVE or P supplement (3 capsules per day in each group) was ingested with water following the first meal of each day.

### 2.3 Assessments of Subjective Statuses

In the efficacy assessment, we had previously decided to perform the per-protocol analysis using a population with a subjective feeling score of a question item “easily get tired” ≥ 2 that displayed little improvement by placebo ingestion during the run-in period, and then conducted the subgroup analysis in subjects 40 years of age and over.

### 2.4 Safety Assessment

To evaluate the safety of SVE supplementation, the incidence of adverse events of each group from 0 through 8W were compared statistically. The study investigator determined whether or not observed adverse events were related to the ingestion of test supplements.

### 2.5 Determination of Anti-oxidative Capacity in Low-Density Lipoprotein (LDL)

LDL oxidative susceptibility was measured using a conventional method (Kurihara et al., 2003). Blood samples were collected into tubes containing ethylenediaminetetraacetic acid (EDTA) from subjects in the morning after an overnight fast. Plasma samples were then stored frozen at –80°C until the LDL oxidation assay. After a plasma sample was adjusted to a specific gravity with KBr (Wako Pure Chemical, Japan), the LDL fraction was subsequently obtained by ultracentrifugation at 100 000 rpm for 40 min. The protein concentration in the LDL fraction was determined using a protein assay kit (Bio-Rad, USA) and adjusted to 70 μg/mL with phosphate-buffered saline. 2,2’-Azobis(4-methoxy-2.4-dimethyl valeronitrile) (V70, Wako Pure Chemical, Japan) was added to a final concentration of 400 μM, followed by incubation at 37°C. Absorbance at 234 nm was measured using a spectrophotometer (UV-2550, Shimadzu, Japan), and the lag time until the start of conjugated diene formation was measured.

### 2.6 Statistical Analysis

All results are presented as the mean ± standard error of mean (SEM). When significant differences were detected by Freidman’s test compared to baseline (0W) scores of the questionnaire data, a Wilcoxon’s signed-rank sum test (with Bonferroni correction) was performed. Intergroup comparisons of questionnaire data were performed using the Mann-Whitney U test for 0, 4, and 8W data. When significant differences were detected by repeated measures analysis of variance compared to baseline (0W) scores of the LDL oxidation lag time, a Dunnett’s post-hoc test was performed. Intergroup comparison of the LDL oxidation lag time was performed using an unpaired Student’s *t*-test for 0, 4 and 8W data. Regarding adverse events, the number of subjects and adverse events were tested using the chi-square test of independence. A p value of < 0.05 was considered significant for all statistical tests.

## 3. Results

### 3.1 Subject Characteristics

Three hundred and five subjects completed the study, while 4 people dropped out due to various reasons (treatment of underlying disease, hospitalization, personal situation, and violation of the study protocol). In the efficacy assessment, 111 subjects were used for the per-protocol analysis with individuals excluded for the following reasons: 192 subjects (92 in the SVE group, 100 in the P group) lost fatigue status (subjective status score of a question item “easily get tired” < 2 at 0W) and/or significant improvements with a large placebo effect in fatigue status by placebo ingestion during the run-in period (change in subjective feeling score of a question item “easily get tired” ≥ 2), and 2 subjects (all in the P group) ingested an insufficient amount of capsules. The background characteristics in the subjects for efficacy assessment are shown in Table 1. These data showed no significant difference between SVE group (n = 60) and P group (n = 51).

**Table 1:** Background characteristics of subjects in the per protocol analysis

	Placebo	Sesame lignans and vitamin E	p value
Number	51	60	-
Gender	Male 15, Female 36	Male 17, Female 43	-
Age (years)	42.7 ± 0.9	42.9 ± 1.0	N.S.
Height (cm)	160.9 ± 1.1	161.5 ± 1.2	N.S.
Weight(kg)	59.0 ± 2.5	60.9 ± 2.4	N.S.
BMI (kg/m^2^)	23.0 ± 0.6	23.6 ± 0.5	N.S.
Subjective status score (Easily get tired)	2.73 ± 0.09	2.53 ± 0.08	N.S.

Values are shown as mean ± SEM.

There were no significant differences between groups.

### 3.2 Assessment of Subjective Statuses

Table 2 shows the efficacy assessment of subjective statuses regarding fatigue, sleep, and physical appearance using the 111 subjects in the per-protocol analysis. The total status score of fatigue significantly improved in both the SVE and P groups. The change from 0 to 8W in the SVE group appeared to be greater than that in the P group, although a significant difference was not observed between the groups (SVE -3.22 ± 0.50 vs. P -2.92 ± 0.55). In the efficacy assessment of the subjective statuses regarding sleep and physical appearance, each status score was significantly improved in only the SVE group, although no significant difference was observed between the groups in their change from 0 to 8W (in Sleep: SVE -1.13 ± 0.31 vs. P -0.49 ± 0.29; in physical appearance: SVE -0.80 ± 0.24 vs. P -0.33 ± 0.28).

**Table 2:** The efficacy assessment of subjective satuses in the per-protocol analysis

Status	Group	Score				

		0W	4W		8W	
Fatigue total score	P	13.61 ± 0.49	11.73 ± 0.71	**	10.69 ± 0.63	**
	SVE	12.95 ± 0.39	10.60 ± 0.47	**	9.73 ± 0.46	**
Sleep total score	P	4.02 ± 0.39	3.73 ± 0.43		3.53 ± 0.41	
	SVE	3.80 ± 0.37	3.12 ± 0.31		2.67 ± 0.30	**
Physical appearance total score	P	5.45± 0.31	5.20 ± 0.30		5.12 ± 0.30	
	SVE	5.37 ±0.28	5.10 ± 0.25		4.57 ± 0.23	**

Status scores are shown as mean ± SEM (P, n = 51; SVE, n = 60).

Significant difference from 0W:

** p < 0.01. There were no significant differences between groups.

### 3.3 Subgroup Analysis of Subjective Statuses

**Figure 1:**
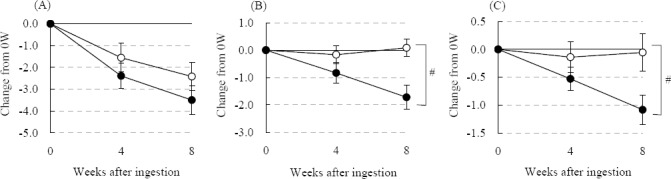
Changes in the total status scores of fatigue (A), sleep (B), and physical appearance (C) in 72 subjects aged 40+ years

A subgroup analysis of subjective statuses was performed based on 72 subjects 40 years of age and older (36 in the SVE group, 36 in the P group). The results are shown in Table 3. The total fatigue status score significantly improved at 4W in the SVE group, and significantly improved at 8W in both the SVE and P groups, although no significant difference was observed between the groups. The total status score of sleep significantly improved at 8W only in the SVE group, and was significantly different between the SVE and P groups. The total status score of physical appearance significantly improved at 8W only in the SVE group, but was not significantly different between the SVE and P groups. As shown in Figure 1, the changes in total status scores for fatigue, sleep, and physical appearance in the SVE group were all improved. Moreover, significant differences were observed in sleep and physical appearance (Figure 1B, 1C).

**Table 3:** The efficacy assessment of subjective statuses using 72 subjects aged 40+ years

Score	Group	Score		

		0W	4W	8W
Fatigue				
Total score	P	13.47 ± 0.60	11.92 ± 0.82	11.06 ± 0.75 **
	SVE	13.42 ± 0.54	11.03 ± 0.62 **	9.92 ± 0.59 **

Easily get tired	P	2.69 ± 0.10	2.19 ± 0.18 *	1.92 ± 0.16 **
	SVE	2.50 ± 0.11	1.97 ± 0.15 **	1.75 ± 0.15 **
Can’t get rid of fatigue	P	2.31 ± 0.15	1.94 ± 0.19	1.69 ± 0.16 *
	SVE	2.22 ± 0.14	1.67 ± 0.14 **	1.64 ± 0.14 **
Feeling of fatigue	P	1.92 ± 0.17	1.69 ± 0.18	1.44 ± 0.18
	SVE	1.72 ± 0.14	1.33 ± 0.14 *	1.14 ± 0.11 **
Feeling languid	P	2.19 ± 0.16	1.97 ± 0.18	1.69 ± 0.18 *
	SVE	2.06 ± 0.15	1.67 ± 0.16 *	1.42 ± 0.13 **
Eyestrain	P	1.58 ± 0.16	1.39 ± 0.15	1.72 ± 0.17
	SVE	1.89 ± 0.13	1.56 ± 0.15	1.36 ± 0.12 *
Vigor	P	2.78 ± 0.14	2.72 ± 0.16	2.58 ± 0.16
	SVE	3.03 ± 0.13	2.83 ± 0.14	2.61 ± 0.17 **

Sleep				
Total score	P	3.78 ± 0.47	3.61 ± 0.49	3.86 ± 0.47

	SVE	3.97 ± 0.47	3.14 ± 0.35	2.25 ± 0.30 ** ##
Light sleeping	P	1.08 ± 0.19	1.19 ± 0.20	1.19 ± 0.20	
	SVE	1.33 ± 0.17	1.08 ± 0.15	0.81 ± 0.13 *
Awaken feeling unrested	P	1.86 ± 0.20	1.61 ± 0.22	1.61 ± 0.18
	SVE	1.56 ± 0.16	1.28 ± 0.14	1.00 ± 0.14 ** ##
Difficulty in falling asleep	P	0.83 ± 0.15	0.81 ± 0.16	1.06 ± 0.19
	SVE	1.08 ± 0.20	0.78 ± 0.14	0.44 ± 0.11 * #

Physical appearance				
Total score	P	5.11 ± 0.36	4.97 ± 0.34	5.06 ± 0.37

	SVE	5.67 ± 0.36	5.14 ± 0.32	4.58 ± 0.29 **
Skin in bad condition	P	0.92 ± 0.16	1.00 ± 0.16	1.06 ± 0.14
	SVE	1.08 ± 0.15	1.06 ± 0.15	0.86 ± 0.13
Poor hair luster/resilience	P	1.33 ± 0.21	1.19 ± 0.17	1.36 ± 0.19
	SVE	1.36 ± 0.18	1.11 ± 0.14	0.86 ± 0.11 **
Youthfulness	P	2.86 ± 0.14	2.78 ± 0.16	2.64 ± 0.16
	SVE	3.22 ± 0.12	2.97 ± 0.14	2.86 ± 0.15 **

Status scores were shown as the mean ± SEM (P, n = 36 [Male 13, Female 23]; SVE, n = 36 [Male 12, Female 24]).

Significant difference from 0W:

* p < 0.05

** p < 0.01, or from the P group:

# p < 0.05

## p < 0.01.

In the assessment of each questionnaire item, the SVE group showed significant improvements with regard to all 6-question items regarding fatigue at 8W after ingestion, and the P group showed significant improvements with regard to 3 items. The change in “eye strain” from 0 to 8W was significantly different between the groups (SVE -0.53 ± 0.17 vs. P 0.14 ± 0.18, p < 0.01). The SVE group showed significant improvements with regard to all 3 sleep status question items at 8W after ingestion, whereas the P group showed no significant improvements on any of the items. Significant differences were observed between the groups for the question items “awaken feeling unrested” and “difficulty in falling asleep.” The changes in “light sleeping” and “difficulty in falling asleep” from 0 to 8W were also significantly different between the groups (in “light sleeping”: SVE -0.53 ± 0.17 vs. P 0.11 ± 0.13, p < 0.01; in “difficulty in falling asleep”: SVE -0.64 ± 0.20 vs. P 0.22 ± 0.16, p < 0.01). In the assessment of physical appearance status questions, the SVE group showed significant improvements with regard to “poor hair luster/resilience” and “youthfulness” 8W after ingestion, whereas the P group showed no significant improvements on all 3 items. No significant differences in “poor hair luster/resilience” and “youthfulness” were observed at 8W between treatment groups. However, the change in “poor hair luster/resilience” from 0 to 8W was significantly different between groups (SVE -0.50 ± 0.15 vs. P 0.03 ± 0.18, p < 0.05).

Each total status score was determined from the score of each item assigned in fatigue, sleep, and physical appearance as described in the Materials and Methods. Solid circles indicate the score of the SVE group (n = 36 [Male 12, Female 24]), and open circles correspond to the P group (n = 36 [Male 13, Female 23]). Values are shown as the mean ± SEM. Significant difference from the P group: #, p < 0.05.

### 3.4 Oxidative Stress (LDL Oxidative Susceptibility)

**Figure 2:**
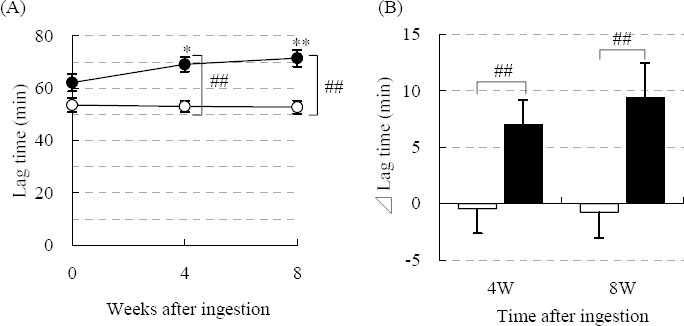
Changes in LDL oxidative susceptibility

Figure 2 shows the LDL oxidative susceptibility of the subjects (14 in the SVE group, 11 in the P group) who were assigned to blood collection at 0W, in advance, and included in the per-protocol population. No significant change in the LDL oxidation lag-time was observed in the P group. However, the LDL oxidation lag-time of the SVE group was significantly prolonged in proportion to ingestion time (Figure 2A), and the change in the LDL oxidation lag-time in the SVE group from 0W was also significantly greater compared to that of the P group at 4 and 8W (Figure 2B).

LDL oxidation lag-times were measured as described in the Materials and Methods. (A) The time-course of LDL oxidation lag-time. (B) Change in lag-time 4W and 8W after ingestion. Solid circles or bars indicate the score of the SVE group (n = 14 [Male 4, Female 10]), and open circles or bars correspond to the P group (n = 11 [Male 3, Female 8]). Values are shown as the mean ± SEM. Significant difference from 0W: *, p < 0.05, **, p < 0.01, or from the P group: #, p < 0.05, ##, p < 0.01.

### 3.5 Safety

The safety analysis of SVE supplementation was performed using the 309 subjects that participated in this study according to the intention-to-treat (ITT) protocol analysis. There was no significant difference in the incidence of adverse events between the SVE and P groups (data not shown), indicating that no side effects relating to the ingestion of SVE capsules were observed.

## 4. Discussion

We evaluated the effect of SVE supplementation on subjective statuses in healthy humans with feelings of daily fatigue. In the efficacy assessment, all the total status scores corresponding to fatigue, sleep, and physical appearance were significantly improved by SVE supplementation in the per-protocol analysis, although the difference from the P group was not significant (Table 1). In the subgroup analysis, subjective statuses of sleep and physical appearance were significantly improved and subjective fatigue status showed a tendency for improvement compared to the placebo (Figure 1). Here, the efficacy of SVE supplementation was more obvious compared to the results of the per-protocol analysis, indicating that the supplementation of SVE is more useful for individuals aged 40 and over experiencing daily fatigue.

We confirmed that SVE supplementation improved the LDL oxidative susceptibility. We have also previously reported that the sesame lignans, sesamin and episesamin, are converted into highly potent anti-oxidative catechol-type metabolites *in vivo* (Nakai et al., 2003) and might act as effective pro-inhibitors of vigorous exercise-induced serum lipid peroxidation in mice and humans (Ikeda et al., 2003; Kiso, 2004). Tomimori et al. (2013) reported that SC-1 and EC-1, catechol-type metabolites of sesame lignans, were detected in human plasma by SVE supplementation. In addition Rogi et al. (2011) reported that sesamin reduced the blood cholesterol level synergistically with α-tocopherol (a ratio of sesamin: α-tocopherol = 1:5 (w/w)) in rats fed a high-cholesterol diet and it enhanced α-tocopherol level not in the serum but in the liver. On the other hand it has been reported that high-dose α-tocopherol supplementation in humans decreases the susceptibility of LDL to oxidation. Jialal, Fuller, and Huet (1995) evaluated the effect of α-tocopherol supplementation on LDL oxidative susceptibility when it was ingested at 60–1200 IU/day for 8 weeks. They showed that the minimum effective dose of α-tocopherol was 400 IU/day, which was much larger than the dosage of VE used in this study. Therefore, it is possible that the improvement in anti-oxidative capacity by SVE supplementation depends on sesame lignans and in part on VE. Further elucidation is necessary to clarify the combination effects of sesame lignans and VE.

A correlation between the level of accumulated fatigue and oxidative stress has been reported. Vecchiet et al. (2003) found that patients with CFS showed worse oxidative stress *in vivo*, including enhanced LDL oxidative susceptibility and thiobarbituric acid reactive substances (TBARS). The level of fatigue in CFS correlates with the blood concentration of 8-isoprostane, a lipid peroxidation marker, indicating that oxidative stress is an important factor associated with fatigue (Kennedy et al., 2005). Eldadah (2010) reported that a number of people felt that they tire easily with increasing age. Estryn-Behar et al. (1990) also found that sleep status changes with age, and that individuals aged 40 and over are especially dissatisfied with their sleeping patterns. Furthermore Niwa et al. (1990) showed that the induction of SOD activity declined with age beginning in the fifth decade, suggesting that anti-oxidant capacity in human body began to fall between 40 and 50 years of age. These might explain why SVE supplementation was more effective in the individuals aged 40 and over. Oxidative damage also contributes to the pathogenesis of various diseases such as cancer, atherosclerosis, and lifestyle-related disease. Aging and its related diseases are caused by free radical-induced damage to cellular macromolecules and the inability of cells to counterbalance these changes by endogenous anti-oxidant defenses (Datta, Sinha, & Chattopadhyay, 2000). Therefore, reduced oxidative stress by SVE supplementation may exert beneficial effects not only on fatigue but also on health in general.

As shown in Table 3, SVE supplementation was most effective at improving sleep status in this study. It is well known that sleep is important for recovery from physical and mental fatigue. Sundelin et al. (2013) reported fatigue is aggravated by sleep deprivation. Kunert et al. (2007) demonstrated that night-shift nurses have a lower quality of sleep and more severe fatigue than day-shift nurses. Poor sleep results in a significantly increased risk of morbidity and mortality (Mallon, Broman, & Hetta, 2002; Mallon, Broman, & Hetta, 2005). In general, physical appearance is also affected by an individual’s quality of sleep and level of fatigue. In the present study, the scores of all 6-item questions assigned to fatigue status were significantly improved in the SVE group, suggesting that improved sleep and physical appearance might be related to the improvement of fatigue status by SVE supplementation.

There are some limitations to the present study. First, we could not completely remove a placebo effect from the subjective fatigue status. A number of studies have been designed to minimize this problem. Ishizaki, Hisano, Umeki, Kuroda, & Hayabuchi (2005) clearly evaluated anti-fatigue effects by excluding subjects strongly affected by the placebo effect. We scheduled a run-in period prior to the parallel-group study in order to minimize placebo effects and to determine which subjects required exclusion from the per-protocol analysis. Nevertheless, the residual placebo effect was observed only in the subjective status of fatigue in this study. This result might reflect the subjects’ knowledge of the study’s objective, which was revealed by the informed consent document. Second, the evaluation of anti-oxidative capacities was performed in the restricted subjects with small sample size. Third, we cannot estimate the contribution of each ingredient, as we carried out this human study using a supplement including sesame lignans and VE. Intervention trials would be needed to overcome these limitations.

## 5. Conclusion

Supplements containing sesame lignans (sesamin/episesamin = 1/1) and VE have been ingested for more than 20 years in Japan for the purpose of health maintenance and we confirmed the safety for the 5-fold doses of supplementation in a human study (Tomimori et al., 2013). In the present study we confirmed that no adverse events relating to SVE supplementation were observed. For the first time, we demonstrated that the supplementation of sesame lignans (sesamin/episesamin = 1/1) with VE could significantly improve subjective fatigue-related status and anti-oxidative capacity particularly in middle-aged and elderly people experiencing feelings of daily fatigue. These results suggest that sesame lignans with VE supplementation is safe and useful for alleviating daily fatigue and oxidative stress.
